# Mapping the evidence on occupational exoskeleton use for the workforce in healthcare, social care, and industry: A systematic scoping review

**DOI:** 10.1017/wtc.2025.10033

**Published:** 2025-10-28

**Authors:** Shilpy Bhat, James Gavin, Martin Warner, Michelle Myall

**Affiliations:** 1 School of Health Sciences, University of Southampton, Southampton, Hampshire, United Kingdom; 2 NIHR Applied Research Collaboration Wessex, Southampton, United Kingdom

**Keywords:** exoskeletons, exosuits, industry, human–robot interaction, performance characterisation, biomechanics

## Abstract

Musculoskeletal disorders remain a leading occupational health challenge in physically demanding sectors such as healthcare, social care, and industry. Exoskeletons – wearable devices designed to mitigate physical strain are increasingly explored as potential solutions; however, factors affecting their adoption in real-world settings remain underexplored. This novel scoping review systematically maps the existing evidence on the application of commercially available exoskeletons within real and simulated work environments, focusing on usage patterns, user experiences, and factors influencing implementation.

Following the Joanna Briggs Institute methodology for scoping reviews, a systematic literature search was conducted across the Web of Science, Scopus, CINAHL, PsycINFO, and MEDLINE, with an initial search in May 2023 and an update in May 2024. Forty-nine papers met the inclusion criteria based on the Population, Concept, and Context (PCC) framework. Data were extracted using a standardized form and synthesized descriptively, thematically, and through content analysis. Results are presented in narrative, tabular, and conceptual map formats.

Exoskeletons were used most frequently in industry (manufacturing) and perioperative care (healthcare). Although, the devices reduced muscle load during repetitive or static tasks, adoption was constrained by discomfort and fit challenges, thermal burden, and limited usability in dynamic settings. Thematic analysis revealed how user experiences were shaped by professional identity, task compatibility, organizational support, and social norms. A conceptual map synthesized sector-specific and cross-sectoral barriers and facilitators.

This review highlights the need for inclusive, context-sensitive, and longitudinal research to support safe, acceptable, and effective exoskeleton adoption and implementation across diverse occupational environments.

## 1. Introduction

Musculoskeletal disorders (MSDs), which affect the back, joints, and limbs, represent a leading occupational health challenge worldwide and are closely linked to the physical demands of work-related tasks (Health and Safety Executive, 2023; WHO/ILO Joint Estimates, 2021). In Great Britain, MSDs make up 27% of work-related ill health and contribute to around 21% of lost working days each year (Health and Safety Executive, 2023). Sectors with high manual-handling demands, such as healthcare, social care, and construction, have particularly high injury rates. For example, in health and social care the incidence rate exceeds 1,350 cases per 100,000 workers due to the physical demands of tasks like lifting, repositioning and assisting with mobility in hospitals, care homes, and domiciliary settings (Health and Safety Executive, 2023). These risks remain even after decades of mandatory training and use of equipment (Health and Safety at Work etc. Act 1974 (1974); Health and Safety Executive, 1992 [MHOR]; 2014), showing the need for more innovative and complementary approaches to reduce MSDs.

One such innovation is the occupational or industrial exoskeletons, wearable robotic devices that augment human performance by reducing physical strain during demanding tasks (de Looze et al., 2016). These range from rigid models to soft and elastic versions known as exosuits (Lowe et al., 2019). Biomechanical research continues to highlight the potential of back-support exoskeletons in reducing lower-back muscle activity during repetitive and physically demanding tasks. However, much of this research has been done in laboratories with homogeneous groups, like healthy young men (Kermavnar et al., 2021). Although promising, these results may not capture the dynamic, unpredictable environments of real workplaces (Crea et al., 2021). While recent advancements in wearable robotics, such as soft, active exoskeletons that adapt to user intention, have increased the flexibility and sophistication of these devices (Nasr et al., 2023; Zhou et al., 2021), their real-world use remains limited. Much of the published literature focuses on technical validation or laboratory trials rather than implementation in everyday practice (De Bock et al., 2022).

Interest in using exoskeletons within healthcare has increased (O’Connor, 2021; Vallée, 2024), especially as healthcare systems globally contend with aging workforces, rising physical demands, and post-pandemic staffing pressures. In social care, similar pressures exist. Many manual handling tasks require “double-up care,” meaning two carers working together, further straining workforce capacity. A review of over 12,000 cases by 53 English local authorities found that 80% still required two carers, despite initiatives aimed at reducing this need (Whitehead et al., 2022). These persistent workforce and logistical challenges have fueled similar interest in solutions that can support carers physically without compromising care quality or safety, including solutions for lone work. Policymakers have also recognized the need for enhanced workplace support systems and have proposed reforms through initiatives like the NHS Digital transformation program (NHS Digital, 2022).

Yet, despite the rising interest in healthcare and social care, the literature on exoskeletons remains dominated by industrial and engineering perspectives. High-risk manual handling tasks are also prevalent in sectors like construction (Li and Ng, 2017), manufacturing (Fox et al., 2020), and the automotive industry (Pinho JP et al., 2020), where ergonomic solutions have been widely explored. Although there are clear parallels in task demands across these domains, evidence from industrial contexts is rarely cross-referenced with research in health and care settings. Understanding how implementation insights from industry might transfer to healthcare or social care contexts remains underexplored, limiting potential scale-up and wider uptake.

In the UK, healthcare is centrally funded through the National Health Service (NHS) and delivered by clinical staff in hospital and primary care settings. Social care, by contrast, is commissioned by local authorities or private providers, regulated separately (Care Quality Commission, 2023), and delivered by a 1.6-million-strong workforce (Skills for Care, 2023). These workers often operate alone in people’s homes or small residential care environments, with minimal clinical infrastructure or access to supervision. These distinctions have profound implications for how technologies like exoskeletons are introduced, evaluated, and used in practice. By differentiating between healthcare and social care, this novel scoping review provides a more context-sensitive analysis. By contrast, “industry” in this review serves as a collective term consisting of non-care settings such as manufacturing, construction, agriculture, and logistics. Grouping these sectors under a common industrial framework allows identification of shared themes across physically demanding, non-care occupations. Sector-specific characteristics, for example, differences between automotive assembly and agriculture, are still recorded and discussed, but the umbrella term helps distill transferable insights.

Furthermore, this review prioritizes studies conducted in real-world environments or high-fidelity simulations, focusing on how exoskeletons perform during routine tasks. This emphasis on ecological validity supports an improved understanding of practical feasibility and user experience. The review also synthesizes implementation barriers and facilitators, culminating in a conceptual map designed to inform future policy and practice. These insights are intended to support evidence-based decision-making across research and sector-specific innovation.

## 2. Review aim

To map the current evidence on the use of exoskeletons among the workforce in healthcare, social care, and industry, accounting for qualitative, quantitative, and multiple-methods research.

## 3. Review objectives

1. To explore how exoskeletons are used to perform manual handling tasks and by whom in healthcare, social care, and industry.
2. To characterize the experiences of users of exoskeletons in healthcare, social care, and industry.
3. To identify factors influencing the adoption and implementation of exoskeletons in healthcare, social care, and industry.

## 4. Study design and methods

A scoping review was conducted following the Joanna Briggs Institute (JBI) framework (Peters et al., 2022), selected for its suitability in addressing broad, exploratory questions and accommodating diverse evidence types, including qualitative, quantitative, and mixed-methods studies (Munn et al., 2018). This approach was particularly appropriate given the limited research on exoskeletons in health and social care at the time of the review (Kermavnar et al., 2021), and the need to map evidence across multiple sectors and methodologies. A review protocol was developed and internally reviewed to ensure methodological rigor, although it was not published, as protocol registration is not mandatory for scoping reviews.

The review followed the JBI nine-stage process (Table 1), which structured the design, evidence selection, data extraction, and the synthesis phase of the review.

**Table 1.** JBI’s nine-stage framework for conducting scoping reviews

Steps	Components of the review process
1	Defining and aligning the objectives and research questions
2	Developing and aligning inclusion criteria with the objectives and questions
3	Describing the planned approach to evidence searching and selection
4	Searching for evidence
5	Selecting the evidence
6	Extracting the evidence
7	Analyzing the evidence
8	Presentation of the results
9	Summarizing the evidence, making conclusions, and noting findings implications

### 4.1 Eligibility criteria

Eligibility was guided by the Population, Concept, and Context (PCC) framework recommended by JBI (Peters et al., 2021). Table 2 outlines the full inclusion and exclusion criteria. Research involving healthy adults aged eighteen and above, trialing or using commercially available exoskeletons in work-related settings, including healthcare, social care, and industrial environments, was included. Studies were excluded if they focused on military, therapeutic, or rehabilitation applications or involved workers with preexisting neuromuscular or musculoskeletal disorders. Although scoping reviews often include a wide range of sources, this review prioritized peer-reviewed primary research to enhance the consistency and manageability of synthesis.

**Table 2.** Inclusion and exclusion criteria

Criteria	Inclusion	Exclusion
Population	Healthy adult workers (aged ≥18 years) working in healthcare; social care; and industry without preexisting WRMSDs, from Industrial workers in healthcare, manufacturing, construction, logistics, and stakeholders (e.g., safety managers)	Research involving animals, as well as those focusing on health and social care professionals or industrial workers with a history of neuromuscular disorders, musculoskeletal disorders, or recurring injuries
Concept	Studies on commercially available exoskeletons/exosuits for occupational tasks (both active and passive) Research on adoption; implementation; and user experiences (subjective and objective)	Studies focused on military applications, medical rehabilitation, motor relearning, or unrelated commercial uses Assistive devices for restoring function or therapeutic training tools
Context	Conducted in healthcare (e.g., surgical; patient handling); social care (e.g., caregiving); and industrial (e.g., manufacturing; logistics; construction) settings Prioritizing firsthand practical work over software-based simulations or mechanical outcomes.	Studies in nonwork-related settings (e.g., recreational or home use)
Types of evidence sources	Empirical research (qualitative; quantitative; multiple methods; and mixed methods)	Protocols, reviews to avoid duplication, grey literature (theses); letters to editors; notes; news reports; conference abstracts; posters and publications focused solely on exoskeleton design and policy documents

Abbreviations: WRMSD, work-related musculoskeletal disorder.

**Figure 1.**
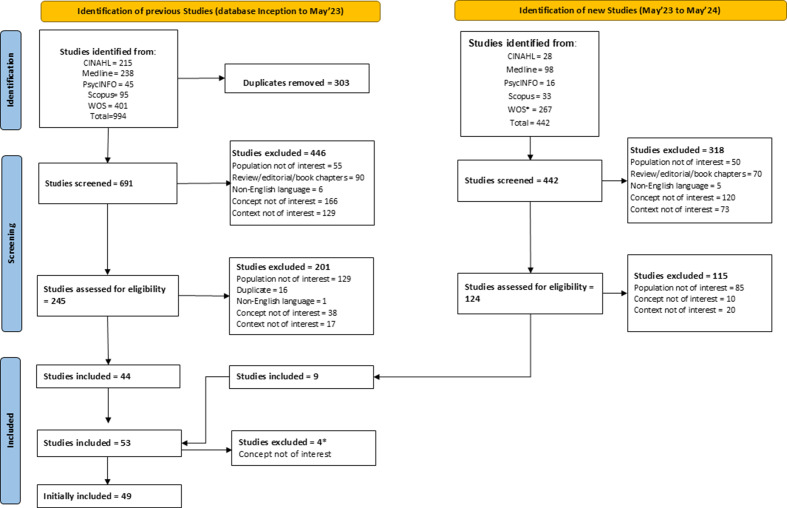
PRISMA 2020 flow diagram for updated systematic reviews which included searches of databases. Adapted from: Page MJ et al., BMJ 2021;372:n71. doi: http://doi.org/10.1136/bmj.n71. **Note:** *Four studies were excluded post-peer review following reappraisal against the Concept domain (i.e., lack of clarity on the commercial availability of the exoskeleton).

To improve transparency during screening, the PRISMA flow diagram (Figure 1) includes the exclusion categories “population not of interest,” “concept not of interest,” and “context not of interest.” These correspond to the PCC framework and were used to classify records that involved ineligible populations, unrelated interventions or outcomes, or nonoccupational settings.

### 4.2 Search strategy

An initial exploratory search was conducted in January 2023 by the first reviewer (SB) to identify key terms, refine the search strategy, and access foundational literature. Key terms included “exoskeleton,” “exosuit,” “wearable robot,” “workforce,” “industry,” “healthcare,” and “social care.” Boolean operators (AND, OR) and proximity operators (NEAR, WITHIN) were used to ensure a systematic and targeted approach.

The final strategy was adapted for five databases: Web of Science, Scopus, CINAHL Plus, PsycINFO, and MEDLINE (all via EBSCOhost, except Scopus and Web of Science), selected for their relevance to exoskeleton applications across care and industry sectors. Full details, including search strings and operators, are provided in Appendix 1 (Supplementary Materials).

To capture more recent evidence, the search was updated on May 30, 2024, and automated alerts were set to identify new publications. A PRISMA 2020 flow diagram (Figure 1) documents the search process, screening decisions, and reasons for exclusion.

### 4.3 Evidence selection and screening

The database search yielded 994 citations. After deduplication in EndNote 20 (Clarivate Analytics, PA, USA), 691 records remained for title and abstract screening. A pilot test on 25 randomly selected titles was conducted on May 15, 2023, by the first reviewer (SB) and a second reviewer (JG) to refine and calibrate inclusion criteria.

Title and abstract screening excluded 446 records. A random 20% sample (*n* = 138 studies) was independently reviewed by the JG in July 2023, following consultation with a subject specialist librarian. High inter-reviewer agreement was achieved, with discrepancies resolved through discussion. Of 245 full-text articles screened by SB, all exclusions were independently reviewed by JG to ensure accuracy. An updated search in May 2024 identified nine additional papers. While 53 studies initially met the inclusion criteria, 4 were excluded after peer review due to misalignment with the Concept domain, resulting in a final total of 49 studies for analysis.

### 4.4 Data extraction

Following JBI methodology (Peters et al., 2020), data extraction was conducted in multiple stages. A standardized data extraction grid was developed in Microsoft Excel® to systematically capture study characteristics and review relevant data. Key fields included citation details, study design, country, exoskeleton type [brand/model and body region supported], and sector [healthcare, social care, or industry]. Where specific brand names were not reported, inclusion was based on detailed descriptions indicating commercial availability and occupational use. Studies were also categorized by context:

- **Field simulation / laboratory:** A controlled field environment simulating real-world occupational tasks.
- **Field / real-world:** Natural work settings without experimental manipulation.

Charted data informed the descriptive mapping for Objective 1 and supported the development of Table 3. For Objectives 2 and 3, qualitative findings were inductively coded into Excel worksheets. Extraction was conducted by the first reviewer [SB], with verifying through discussion with the review team [JG, MM, MW]. Microsoft Excel® was used throughout the extraction and analysis process, enabling structured charting, coding, and synthesis within a unified platform.

**Table 3.** Characteristics of the source of evidence

Study ID	[Author, year]; Country	Type of study; study design	Participants; sample size [male/female]	Industry	Type and brand	Measurement tool [s]
1	(Liu et al., 2018); USA	Prospective randomized crossover study; multiphase	*n* = 20 [gender unspecified]; general surgery residents and attendings	Healthcare [laparoscopic surgery]	Passive arm support exosuit; Levitate AIRFRAME™	MMDT [Minnesota Manual Dexterity Test]; PPDT [Purdue Pegboard Dexterity Test]; FLS [Fundamentals of Laparoscopic Surgery]
2	(Ivaldi et al., 2021); France	Pilot study; simulation-based testing and ICU field implementation	*n* = 5 [2 M/3 not reported]; ICU healthcare workers [2 deployed in ICU]	Healthcare [ICU; COVID–19 prone positioning tasks]	Passive back support; Laevo v1, CORFOR, BackX, CrayX [only Laevo used in field setting]	Xsens motion capture; ECG [Holter electrocardiogram]; Borg CR–10; Technology Acceptance Questionnaire; Semi-structured feedback
3	(Cha et al., 2019); USA	Qualitative usability study with simulation-based evaluation	*n* = 14 staff members: 7 surgical residents [5F, 2 M]; 4 surgical nurses [1F, 3 M], 3 attending surgeons [1F, 2 M]	Healthcare – perioperative [Operating Room]	Passive arm-support exoskeleton; Levitate AIRFRAME™	FLS; Peg transfer simulation [10-min task]; SUS [System Usability Scale]; Descriptive participant survey on musculoskeletal symptoms
4	(Hwang et al., 2021); USA	Experimental study; Repeated Measures Design	*n* = 20 [3 M/17F]; Professional caregivers	Healthcare -Simulated transfers between a wheelchair and a bed	Passive back-support exoskeleton; FLx ErgoSkeleton; V22 ErgoSkeleton; Laevo V2.5	EMG [Surface electromyography]; SUS; Optical motion capture system
5	(Arnoux et al., 2023); France	Experimental study; Repeated Measures Design	*n* = 7 [6 M/1F]; Workers from the surgical department	Healthcare -Surgery	Passive upper limb exoskeleton; Hapo MS™ [ErgoSanté Technologie, France].	EMG; Borg CR–10 scale; SUS
6	(Turja et al., 2022); Finland	Experimental study; Mixed Methods	*n* = 23; S1: *n* = 16 [1 M/15F]: Nursing students; S2: *n* = 7 [1 M/6F]: Professional nurses	Healthcare -Geriatric care	Passive back-support; Laevo	UTAUT; Survey based on Unified Theory of Acceptance and Use of Technology; video observation
7	(Katsarou et al., 2023); USA	A prospective, observational study	*n* = 9 [8 M/1F]; Interventional radiologists [*n* = 8], vascular surgeon [*n* = 1]	Healthcare – Academic hospital	Passive support; StemRad MD radiation protection system	IMU [Inertial Measurement Unit]
8	(Pinho JP et al., 2020); Brazil	Experimental study; Crossover Design [with and without exoskeletons]	*n* = 2 M/0F; Right-handed automotive industry workers	Automotive manufacturing [simulated tasks in lab near assembly line]	Passive Upper Limb Exoskeleton; ShoulderX [SuitX™]; MATE™ [Comau, Italy] Paexo™ [Ottobock]	EMG [Myotrace 400]; synchronized digital video camera [ELP]; IMUs [GY–521]; Arduino Nano for data transfer
9	(Van Engelhoven et al., 2019); USA	Experimental study; Repeated measures design	*n* = 14 [14 M/0F]; Right-handed workers	Simulated industrial setting [construction/manufacturing-related overhead tasks]	Passive Shoulder support Exoskeleton; SuitX; ShoulderX v1 [arms] and v2 [torso frame]	EMG; Borg CR10; task preference
10	(Siedl and Mara, 2022); Austria	Qualitative Study; Focus group discussions	*n* = 18 [11 M/7F]; Workers engaged in various physical material handling tasks	Food retail, Corporate logistics	Passive and active exoskeletons. Chairless Chair [Noonee]; V22 ErgoSkeleton [StrongArm Tech]; PLAD [PeakWorks]; Paexo Shoulder [Ottobock]	Focus group discussions
11	(Schwerha et al., 2021); USA	Qualitative study; focus group	*n* = 67 [57 M/10F]; diverse roles [line, management, engineering, safety, quality control, HR]	Manufacturing [various SMEs]	Passive exoskeletons: Shoulder support: EksoVest™ [Ekso Bionics], AIRFRAME™ 2.0 [Levitate] Back support: Laevo™ v2.5, BackX AC™ [SuitX] Tool/arm support: Steadicam™ Fawcett Vest + ZeroG2™ Arm [Ekso Bionics]	Focus group discussions; Likert scale probability ratings of adoption; observational feedback on comfort/mobility
12	(Moyon et al., 2019); France	Ecological approach; multistage exploratory	*n* = 17; 11 field: 7 M/4F; 6 labs: 6 M/0F; Workers	Manufacturing – boat manufacturing	Passive Upper Limb Exoskeleton; SkelEx™ [Skelex]	Observational grid [RULA, arm releases]; Open interviews; Self-confrontation interviews; Likert Scales; CR10 Borg Scale
13	(Kim et al., 2019); USA	Qualitative approach	*n* = 26 [gender unspecified]; Construction industry representatives: vice president, project managers/engineers, safety and health managers/directors, and carpenters	Construction	Not used; participants shown descriptions and images of commercially available arm and back-support exoskeletons [brands not specified]	Semi-structured interview guide
14	(Hensel and Keil, 2019); Germany	Field study; 4-week trial	*n* = 30 [30 M/0F]; automotive manufacturing workers	Automobile manufacturing	Passive back-support exoskeleton; Laevo™ v2.5	Likert scales for physical and wearing discomfort; Body Part Discomfort Scale; UMUX Lite; Intention-to-use scale based on TAM2; open-ended user feedback
15	(Gonsalves et al., 2023a, 2023b); USA	Empirical study; multiple methods	*n* = 14 [14 M/0F]; pipe laying workers	Construction	Passive back-support exoskeleton BackX™ [SuitX] v2	Borg CR10 scale; Usability questionnaire [Likert scale]; structured and semi-structured interviews
16	(Bennett et al., 2023); USA	Pilot study; Multiple methods	*n* = 4 [4 M/0F]; Task 1: [4 M/0F] Task 2: [3 M/0F]; Professional construction workers	Construction	Passive exoskeletons; Back-support: HeroWear Apex; Arm-support: Ekso EVO, Hilti EXO–01	IMU motion capture data [XSens]; NASA Task Load Index; Heart rate monitor [Polar H10]; post-use surveys
17	(Pacifico et al., 2023); Italy	Quantitative; case series	*n* = 10 [6 M/4F]; cleaning workers	Cleaning services	Passive upper-limb exoskeleton; MATE™ [COMAU]	Surface EMG [BTS FREE EMG 1000]; Electro goniometer; Borg CR10 [RPE]; Usability Questionnaire [SUS-extended]; Technology Acceptance Model [TAM]
18	(Schwerha et al., 2022); USA	Exploratory field study with repeated exposure [two sessions]; multiple methods	*n* = 15 [14 M/1F]; Experienced workers	Manufacturing [SMEs]	Passive exoskeletons: Arm support: EksoVest™ [Ekso Bionics], AIRFRAME™ v2.0 [Levitate] Back support: Laevo™ v2.5, BackX™ [SuitX]	Questionnaire [11-point Likert scale, open-ended questions]
19	(Pinho JP and Forner-Cordero, 2022); Brazil	Experimental study; single-session crossover study	*n* = 14 [12 M/2F]; Right-handed automotive workers	Automotive manufacturing [simulated tasks in lab near assembly line]	Passive upper limb Exoskeleton; ShoulderX v1 [SuitX™]	EMG [MyoTrace 400]; CCI [Co-contraction Index]; Perceived Exertion Scale; Comfort/Safety/Acceptance Questionnaire; Task timing [video-based]
20	(Pacifico et al., 2022); Italy	Case series; mixed in-field and simulated task evaluation	*n* = 7 [7 M/0F]; workers in cabinet production	Manufacturing—cabinet production	Passive shoulder support exoskeleton; MATE™ [COMAU]	EMG; Electro goniometer; Borg CR10; Local Perceived Exertion [LPE]; System Usability Scale [7-pt]; TAM questionnaire [8 constructs]
21	(Ziaei et al., 2021); Iran	Cross-sectional study: multiple methods – Within-subject study designed to compare variables with and without the Ergo-Vest	*n* = 20 [20 M/0F]; waste collectors	Waste management [manual waste collection]	Passive low-back support vest; Ergo-Vest	3DSSPP software; Polar RS400 Heart Rate Monitor Borg’s Scale; RPE; SUS; Custom-made questionnaire
22	(Flor et al., 2021); Portugal	Pilot study	*n* = 23 [gender unspecified] Automotive industry workers; 7 at Workstation [WS] 1, 4 at WS2, 12 at WS3;	Automotive Manufacturing—Press area	Passive back support; Laevo™ v2.56	Borg CR10 scale; Daily Ease-of-Use Questionnaire; PSSUQ [Post-Study System Usability Questionnaire]; Custom Likert scales [discomfort, donning/doffing, and intention to use]
23	(Siedl et al., 2021); Austria	Field study; exploratory	*n* = 58 [26 M /32F]; Supermarket employees	Food retail corporate logistics	Passive exoskeletons [rigid and soft]: Back support [rigid]: Laevo™ V2.5 Arm support [rigid]: Atlas [Exomys] Leg support [rigid]: Daedalus [Exomys] Lumbar support [soft]: Rakunie [Morita] Posture support [soft]: Paexo Soft Back [Ottobock]	Post-trial questionnaire: ITU [Technology Usage Inventory]; Task-technology fit [study-specific scale]; comfort; strain relief; work adaptation
24	(De Bock et al., 2020); Belgium	Multiple methods; field and laboratory-based evaluation with repeated measures	*n* = 4 [4 M/0F]; industrial workers	Automative – European distribution centre of Carglass	Passive shoulder support exoskeletons; ShoulderX™ V2 [SuitX] and Skelex™ V2 [Skelex]	EMG; ECG; accelerometers, RPE [Borg 6–20]; SUS, NASA-TLX; Body Part Discomfort Scale
25	(Thamsuwan et al., 2020); Canada	An experimental study combining controlled laboratory experiments and field-based trials.	*n* = 20; Field: [14 farmers and farmworkers: 13 M/1F]; Lab: 6 females, non-farmers]	Agriculture	Passive back-support exoskeleton; Laevo™ V2.5 [Laevo Exoskelet]	EMG
26	(Iranzo et al., 2020); Spain	Ergonomic assessment	*n* = 12 [11 M/1F]; Employees of the Ford automotive factory	Automotive assembly plant	Passive upper-limb exoskeletons; Levitate AIRFRAME™.	EMG; motion capture
27	(Elprama et al., 2020); Belgium	Quantitative presurvey-based study	*n* = 124 [111 M/13F]; Workers from three companies interested in adopting exoskeletons	Multiple	Back and shoulder exoskeletons; Laevo™; SuitX™ BackX	Survey based on the UTAUT model adapted to the exoskeleton context.
28	(Cardoso et al., 2020); Portugal	Multiphase study; P1: A psychophysical assessment P2: An electromyographic [EMG] assessment	Psychophysical assessment: *n* = 7 [5 M/2F]; EMG assessment: *n* = 5 [2 M/3F]; Workers	Furniture manufacturing	Passive back-support exoskeleton; Laevo™	Borg CR–10 Scale; Likert Scale; VAS; EMG; using wireless biosignals [Plux HUB®]
29	(Marino, 2019); USA	Field trial; single-shift crossover	*n* = 14 [11 M/3F]; Stockers and tyre installers	Wholesale and Retail trade	Passive back-assist exoskeletons; Levitate AIRFRAME™ Shoulder-assist exoskeletons; SuitX™ BackX [models AC and S]	Wearable sensors [GoX Ergo system]; questionnaire [Likert scales, open feedback]
30	(Smets, 2019); USA	Multiphase field trial	P1:*n* = 8 [7 M/1F], P2: *n* = 10 [9 M/ 1F], P3: 4 [3 M/1F]; Automotive assembly operators across three pilot studies	Manufacturing – Automotive assembly	Passive arm support exoskeleton; EksoBionics	Cornell Musculoskeletal Discomfort Questionnaire; usability/fit [custom Likert-scale]; feedback on comfort/thermal; open comments
31	(Gillette and Stephenson, 2019); USA	Field ergonomic assessment with crossover repeated measures	*n* = 6 [4 M/ 2 F]; Workers	Manufacturing	Passive upper-body exoskeleton; Levitate AIRFRAME™	EMG [using Trigno wireless EMG system, Delsys, Natick, MA]; Post-experience Survey
32	(Omoniyi et al., 2020); Canada	Cross-sectional study	*n* = 15 [gender not specified]; Farm workers	Agriculture	Passive back-support exoskeleton; Laevo™ V2.5	Standardized task protocol [lifting, bending]- NIOSH lifting guidelines; Semi-structured interviews
33	(Jorgensen et al., 2022); USA	Experimental, within-subjects design	*n* = 16 [8 M/8F]; aircraft manufacturing workers	Aircraft manufacturing	Passive shoulder exoskeletons; EVO [Ekso Bionics]; Skelex 360XFR [Skelex]; Paexo [Ottobock]	EMG [using Noraxon TeleMyo G2 2400R telemetry system]; Exoskeleton preference and feedback
34	(Kim et al., 2022); USA	18-month longitudinal controlled study (field trial)	ASE group: *n* = 41; (30M / 3W/ 8 not reported); Control: *n* = 83; (M47/ F14/ 22 not reported); Final assembly operators [overhead work]	Manufacturing—automotive assembly	Passive arm-support exoskeleton; EksoVest™ [Ekso Bionics, Inc.]	Custom usability questionnaire (6 items, 0–10 scale); open-ended feedback; intention-to-use query; medical visit records
35	(Kim et al., 2021); USA	18-month longitudinal controlled study (field trial)	ASE group: *n* = 41; (30M/ 3W/ 8 not reported); Control: *n* = 83; (M47/F14/22 not reported); Final assembly operators [overhead work]	Manufacturing -automotive assembly	Passive arm-support exoskeleton; EksoVest™ [Ekso Bionics, Inc.]	CMDQ [Cornell Musculoskeletal Discomfort Questionnaire]; OCRA [Occupational Repetitive Actions Index]; video job analysis, linear mixed models
36	(Kopp et al., 2022); Germany	Prospective methodology and feasibility study (Exoworkathlon Parcours)	“Young expert” workers (*n* not reported here; selected for industrial experience	Multiple- logistics, automotive assembly, welding, and timber construction.	Not specified in detail; multiple CE-marked upper limb and back exoskeletons	EMG [Heart rate monitoring via a smartwatch]; Impedance cardiography; SUS; Borg CR10 Scale; Body Chart for Stress Perception Ratings
37	(Farah et al., 2023); France	Two-phase pilot study (selection + user testing)	*n* = 14 (12F; 2 not specified); nurses	Healthcare [Hospital – (internal medicine, ICU, geriatrics)]	Active lumbar support exoskeleton; ATLAS (JAPET)	Self-administered 20-item questionnaire [clinical, technical, ergonomic, nurse satisfaction, fatigue impact]; developed by a multidisciplinary team
38	(Frost et al., 2024); Denmark	Mixed methods; field trial (5 months), interviews, survey, observation	*n* = 26 (gender not specified); Danish slaughterhouse workers, with additional input from managers	Slaughterhouse [meat processing]	Passive exoskeleton; ShoulderX V4 [SuitX™, USA]	Semi-structured interviews; Borg CR10 scale, on-site observation
39	(Gonsalves et al., 2023a, 2023b); USA	Experimental; multiple methods	*n* = 8 [8M/0F]; concrete workers	Construction – concrete work	Passive back-support exoskeleton BackX™ [SuitX]	CR10 Borg scale; Usability questionnaire; semi-structured interviews
40	(Gonzales et al., 2023); USA	Multiple methods	*n* = 34 [18M/16F]; surgeons, surgical residents, OR nurses, surgical technicians, and central processing technicians [CPTs]	Healthcare – perioperative environments	Passive back support; SuitX™ BackX; HeroWear Apex Exosuit. Passive shoulder support: Paexo Shoulder; Ekso EVO	Nordic Musculoskeletal Disorder questionnaire; IPAQ [International Physical Activity Questionnaire]; qualitative interviews; simulated workflow observation
41	(Wioland et al., 2024); France	Cross-sectional survey design (two campaigns)	Campaign 1: *n* = 181 [94 nonusers, 87 users/ex-users]; Campaign 2: *n* = 78 users; (Gender not specified); workers	Multiple industries – logistics, agri-food, transportation, automotive, and construction	Passive and active exoskeletons; Various models	UTAUT and Situated Acceptance based acceptability; acceptance questionnaire (7 dimensions, 33 items)
42	(Saurio et al., 2023a); Finland	Qualitative field study; involving a three-week trial period	*n* = 8 assistant nurses (gender not specified)	Social care [Elderly care homes]	Passive back-support exoskeleton; Auxivo LiftSuit® 2.0	Pre- and post-interviews user diaries
43	(Saurio et al., 2023b); Finland	Qualitative field study	*n* = 11 [8 assistant nurses; 3 managers] (gender not specified)	Social care [Elderly care homes]	Passive back support exoskeleton; Auxivo LiftSuit®	Pre- and post-interviews, user diaries; individual interviews with managers
44	(Riemer and Wischniewski, 2023); Germany	Qualitative, expert interviews, longitudinal (1 year)	*n* = 4 occupational safety experts (supervising 50 to 400 users each)	Multiple -manufacturing, construction, logistics, healthcare	Passive (rigid) BSEs (PassEXO), Quasi-passive soft BSEs (SoftEXO)	Repeated in-depth expert interviews
45	(Jakob et al., 2023); Germany	Mixed methods; four case studies	CS1: 4 [2M/2F, incl. 1 manager] workers; CS2: 3M farm managers; CS3: 3F workers; CS4: 11 [6F/5M] workers	Agriculture	Passive back support exoskeletons; Hapo MS™ [ErgoSanté, France]; Apex [Herowear, USA]; Liftsuit [Auxivo AG, Switzerland]	Custom-developed Usability and Acceptance Questionnaires; Nordic Questionnaire for Musculoskeletal Disorders; Photo and video documentation
46	(Settembre et al., 2020); France	Early feasibility findings from the ICU simulation phase; the data were later expanded in Ivaldi et al. [Study ID 2]	*n* = 5 [gender: 2M/ 3 not stated]; ICU staff	Healthcare – ICU, Covid–19	Passive back support; Laevo™ v1, CrayX, BackX, CORFOR [tested during simulation]	Narrative summary only [device comparison feedback, feasibility observations]; no structured instruments reported
47	(Elprama et al., 2023); Belgium	Qualitative Study; Design Ethnography, which included fieldwork, participant observation, focus groups, interviews, and surveys	*n* = 160 [145 M/15F] survey respondents; *n* = 30 [29 M/1F] focus group participants; 1 regular EXO user [gender not reported]; Workforce from three companies	Multiple- manufacturing, distribution, and air-conditioning production	Passive exoskeletons; Laevo™ V2 & BackX; ShoulderX [SuitX™]; Skelex V2	Focus groups; individual interviews; co-creation exercises; surveys
48	(Botti et al., 2023); Italy	Preliminary empirical study; controlled field experiment	Task A: *n* = 3 Task B: *n* = 3 [gender not specified]; workers	Industrial manual handling (warehouse/factory)	Passive back-support exoskeleton; Laevo™ V2	Baropodometric sensorized insoles (FlexInFit, Sensor Medica) with 214 sensors per insole
49	(Ferreira et al., 2020); Portugal	Field trial; Prospective interventional	*n* = 88 [gender not specified]; Workers	Manufacturing -Automotive assembly	Passive upper-limb exoskeleton; Skelex MARK 1.3®	Usability questionnaires; daily questionnaires; Technology Usage Inventory [TUI]), Borg RPE scale, 7-point Likert scale (discomfort, utility, intention to use), open-ended comments (frequency analysis)

*Note:*

1. Studies 35 and 34 (Kim et al., 2021, 2022) form part of the same 18-month longitudinal program of research in the automotive sector, with each paper reporting distinct phases of data collection or outcomes.

2. Studies 42 and 43 (Saurio et al., 2023a, 2023b) report on the same field trial [TUEKS field study] of the Auxivo LiftSuit 2.0^®^ in a Finnish care home. The former presents user experiences from interviews and diary data, whereas the latter integrates these insights within a broader human-centered design and implementation context.

3. When multiple reports referred to the same study, for example, Settembre et al., 2020; and Ivaldi et al., 2021, data were extracted only from the most comprehensive source to avoid duplication.

4. Study 36 (Kopp et al., 2022) describes the Exoworkathlon, a controlled simulation/field protocol for evaluating commercial exoskeletons in realistic industrial tasks. It is included for its methodological contribution but does not report on user experience or implementation outcome.

### 4.5 Data analysis and presentation of results

The review followed PRISMA-ScR guidelines (Tricco et al., 2018), with the PRISMA-ScR reporting checklist provided in Supplementary Appendix 2. Consistent with JBI recommendations (Pollock et al., 2023), synthesis combined frequency counts, tabular summaries, and narrative descriptions. Given the differing purposes of Objectives 2 and 3, distinct qualitative synthesis approaches were applied. For Objective 2, which aimed to characterize the experiences of exoskeleton users across sectors, no prior analytic framework was applied. Instead, an inductive, data-driven, thematic analysis (Braun and Clarke, 2006) was used iteratively. SB proceeded through the six phases: familiarization with the data, generating initial codes, searching for themes, reviewing themes, defining and naming themes, and producing the report. It allowed in-depth exploration of users’ perspectives and experiences and identified sector-specific and cross-sector themes. In contrast, Objective 3 focused on identifying factors influencing the adoption and implementation of exoskeletons. Here, basic content analysis was used to inductively code and quantify higher order barriers and facilitators, with each unique barrier or facilitator counted once per study to prevent overrepresentation.

The following section presents the findings of the included studies, structured according to the review’s three objectives.

## 5. Results

### 5.1 Objective 1: To explore how exoskeletons are used to perform manual handling tasks and by whom in healthcare, social care, and industry

Note on study references: Study identifiers (IDs), e.g., [1], [2], refer to specific papers listed in Table 3, which also contains full citation details. Study IDs are used throughout the Results to indicate evidence sources (see Table 3 for citations and mapping).

The review analyzed 49 sources from various publication types. These included journal articles; conference proceedings [2, 8, 22, 23, 28]; e-book chapters [43]; book sections [48, 49]; and one editorial [46]. Key study characteristics are summarized in Table 3; a summary of key trends follows. 13 were conducted in a controlled field, simulation, or laboratory environment to assess occupational tasks using multiple methods [2–4, 8, 9, 19, 20, 31, 33, 36, 39, 40, 46].

#### 5.1.1 Year of publication

Studies were published between 2018 and 2024. One study was published in 2018 [1]. This was followed by 8 studies in 2019 [3, 9, 12, 13, 14, 29, 30, 31]; and 9 in 2020 [8, 24, 25, 26, 27, 28, 32, 46, 49]. In 2021, 7 studies were published [2, 4, 11, 21, 22, 23, 35], and 8 in 2022 [6, 10, 18, 19, 20, 33, 34, 36]. The highest number appeared in 2023, with 14 studies [5, 7, 15, 16, 17, 37, 39, 40, 42, 43, 44, 45, 47, 48]. Two further studies were published in 2024 [38, 41].

#### 5.1.2 Country and setting

The reviewed studies were published across 13 countries, reflecting the global nature of research on occupational exoskeletons. The United States led with 18 studies [1, 3, 4, 7, 9, 11, 13, 15, 16, 18, 29, 30, 31, 33, 34, 35, 39, 40], followed by France (*n* = 6; [2, 5, 12, 37, 41, 46]) and Germany (*n* = 4; [14, 36, 44, 45]). Portugal (*n* = 3; [22, 28, 49]), Italy (*n* = 3; [17, 20, 48]), Finland (n = 3; [6, 42, 43]), and Belgium ([n = 3; 24, 27, 47]) contributed three studies each. Additional studies originated from Austria (*n =2*; [10, 23]),Canada (*n =2*; [25, 32]), Brazil (*n =1*; [8]), Spain (*n =1*; [26]), Denmark (*n =1*; [38]), and Iran (*n =1*; [21]). This distribution highlights the predominance of research from Europe and North America, with smaller but notable contributions from South America and Asia.

The studies were conducted across diverse global contexts and mapped into one of three primary sectors: industry (*n* = 37), healthcare (*n* = 10), and social care (*n* = 2). The majority of studies focused on industrial settings [8–24, 25–36, 38–39, 41, 44, 45, 47–49], reflecting the longstanding interest in exoskeletons for physically demanding roles. Within this category, manufacturing was the most represented sub-sector (*n* = 18; [8, 9, 11, 12, 14, 18–20, 22, 28, 30, 31, 33–35, 44, 47, 49]). Other subsectors included construction (*n* = 4; [13, 15, 16, 39]), logistics and retail (*n* = 5; [10, 23, 24, 29, 41]), agriculture (*n* = 3; [25, 32, 45]), cleaning services [17], waste management [21], and meat processing [38]. Several studies, for example, 27, 36, 41, 44, and 47, spanned multiple industrial subsectors, highlighting cross-domain applicability. In contrast, healthcare settings were represented by 10 studies [1–7, 37, 40, 46], covering diverse environments including perioperative care, surgery, geriatric wards, ICUs, and academic hospitals. Only two studies [42, 43] were situated in social care contexts, both conducted by the same research group in Finland, indicating a limited body of empirical evidence from this sector.

This distribution reflects a strong research focus on the industrial sector, with relatively fewer studies examining healthcare and a notable lack of empirical work in social care, despite its high physical workload demands.

#### 5.1.3 Device type and target users

Forty-nine included studies explored a total of 17 distinct exoskeleton devices, comprising at least 25 distinct model variations. These spanned commercially available passive (≈85%), active, and hybrid or quasi-passive systems [10, 37, 41], with the majority designed for back and upper limb support in manual handling contexts. The Laevo™ series (v1, v2, v2.5) was the most frequently studied family of devices. Upper limb/shoulder exoskeletons such as Levitate AIRFRAME™; SuitX™ BackX and ShoulderX; Eksobionics EksoVest™; and HeroWear Apex™ Exosuit were also prominent. 9 major manufacturers were represented. Some studies investigated multiple devices [11, 18, 23], while others examined a single exoskeleton in field or simulation trials. A small number of studies explored commercially available exoskeletons using descriptive or survey-based methods without direct device testing [13, 36].

The target users spanned a wide range of professions, covering healthcare (i.e., nurses, surgeons, and formal caregivers), as well as industrial workers in manufacturing, construction, automotive, farming, waste management, supermarket logistics, and aircraft manufacturing. Mapping evidence across included studies showed that healthcare and caregiving samples were predominantly female or gender-mixed, while industrial sectors such as automotive, construction, and waste management were almost exclusively male. Several studies did not report gender; however, where specified, industrial cohorts typically comprised 80–100% male participants, whereas nursing and caregiving roles had higher proportions of female participants.

#### 5.1.4 Measurement tools

A range of measurement tools were used to assess exoskeleton outcomes. Surface electromyography (EMG) was reported in studies 4, 5, 8, 9, 17, 19, 20, 24, 25, 26, 28, 31, 33, and 36. The Borg CR10 or similar exertion scales appeared in studies 2, 5, 9, 12, 15, 17, 20, 21, 22, 24, 28, 36, 38, and 39. Usability was commonly measured using the System Usability Scale (SUS) or its adaptations [3, 4, 5, 17, 20, 21, 22, 24, 36, 38], while technology acceptance and intention to use were assessed with frameworks such as the Technology Acceptance Model (TAM) and the Unified Theory of Acceptance and Use of Technology (UTAUT) [6, 17, 20, 21, 27, 34, 41]. Motion capture systems and wearable sensors were used in studies 2, 4, 8, 16, 19, 24, 26, 28, 29, and 48. Qualitative methods such as interviews, focus groups, or diaries were reported in studies 10, 11, 12, 13, 15, 18, 30, 32, 38, 39, 42, 43, 44, 45, 46, 47. Some studies used custom or study-specific questionnaires [e.g., 14, 18, 21, 22, 29, 31, 35, 45, 49], and several combined more than one type of tool. The Visual Analog Scale (VAS) was used to measure perceived pain or discomfort [28], and sensor insoles were used for foot pressure and balance [48].

Measurement tool selection varied by sector. Healthcare studies [1–6, 37, 40] most often combined physiological measures (EMG, Borg CR10), dexterity and simulation-based tests (e.g., MMDT, FLS, PPDT), and standardized usability or acceptance frameworks (SUS, TAM, UTAUT). Two social care studies [42, 43] tended to favor semi-structured interviews and user diaries, while studies in the broader industrial sector like, manufacturing, automotive, construction, agriculture were more likely to incorporate motion capture, wearable sensors, heart rate monitoring, and detailed ergonomic protocols alongside usability assessment. Study ID 36 stands out for its methodological contribution, as it established a standardized framework (Exoworkathlon) employing validated measures such as EMG, Borg CR10, and SUS in realistic scenarios, although it did not itself report empirical user outcomes. Overall, across all sectors, there is a trend in more recent studies, particularly within industry, toward the adoption of multiple or mixed methods designs, which integrate quantitative measurement tools such as EMG, Borg CR10, and the SUS with qualitative approaches (including interviews and direct observation). This reflects a growing recognition that quantitative metrics alone are insufficient to capture the full complexity of user experience and contextual barriers in real-world settings. Nevertheless, reporting was inconsistent: some studies provided detailed tool descriptions and combined methods, while others gave only brief or narrative accounts. This mix of validated scales, custom instruments, and qualitative feedback shows an ongoing lack of standardization in outcome measurements across the field.

### 5.2 Objective 2: To characterize the experiences of users of exoskeletons in healthcare, social care, and industry

This thematic synthesis draws on 49 studies across healthcare, social care, and industry. Five inductively developed themes captured how users made sense of exoskeletons in their working lives, shaped by physical, social, and organizational contexts. While certain experiences were echoed across all sectors, each field revealed distinct expectations and challenges. It is important to note that in social care, evidence was more limited, with two linked primary studies [42, 43] informing these themes.

Table 4 summarizes the key patterns of convergence and divergence in user experience across sectors, providing a comparative overview that frames the detailed thematic analysis that follows.

**Table 4.** Cross-sector comparison of user experiences with exoskeletons in healthcare, social care, and industry

Theme	Healthcare	Social care	Industry
Physical relief and support	Short-term relief noted; undermined by sterility, workflow, and task switching challenges.	Relief reported, especially for older staff; framed as task-specific rather than productivity-driven.	Muscle load reduction confirmed; relief was often conditional and linked to overhead/static tasks; sometimes offset by discomfort.
Comfort and usability	Crucial for task fluidity and hygiene; discomfort increased with prolonged use; shorter trials limited long-term insight.	Discomfort during squatting/showering; device entanglement and hygiene issues noted in shared-use settings.	Comfort was tied to heat, movement restriction, and device design; long-term trials show variable adaptation.
Fit and inclusivity	Gender-based exclusion was evident; poor fit affected legitimacy and usability, especially for female users.	Fit was seen as a manageable irritation; less exclusionary impact; device universality was questioned.	Most consistent barrier; poor adjustability led to withdrawal; rigid designs limited inclusivity.
Social and organizational climate	Support from champions (e.g. surgeons) and leadership was critical; social comfort was influenced by clinical norms and patient perception.	Managerial support and nonintrusive trials were reported to foster quiet acceptance.	Organizational culture and training shaped use; peer dynamics and voluntary use were key for sustained adoption.
Meaning and identity	Ethical and relational concerns were raised; some feared loss of human touch; ambivalence about role disruption.	Exoskeletons were perceived as socially neutral; no stigma or symbolic resistance from users or residents.	Symbolic meaning shaped by peer norms; concerns about appearing weak were noted but outweighed by practical issues.

#### 5.2.1 Physical relief and support: a shared need, sector-shaped interpretation

Across the three sectors, exoskeletons were widely valued for their capacity to reduce musculoskeletal strain, though the meaning and impact of this benefit were deeply context-dependent and shaped by the nature of work in each sector and subsector.

In healthcare, short-term physical relief was most relevant during static or sustained procedures. Surgeons reported up to a 70% decrease in shoulder pain and less fatigue, which enabled task completion without impairing dexterity [1]. ICU staff perceived lower back relief during patient positioning tasks, supported by EMG data [2], while imaging staff reported improved posture and reduced ergonomic risk when using the exoskeleton [7]. In nursing contexts [37], 86% of users reported reduced back pain and fatigue during patient care. These outcomes were often framed not only as personal benefits but as factors contributing to sustained care delivery and clinical performance. However, in real-world nursing and surgical units, the translation of physical relief into routine use was more complex. Participants identified field-level barriers, such as sterility concerns, donning and doffing burden, and incompatibility with rapid task-switching, which reduced the long-term feasibility of adoption [5, 40]. This illustrates how even recognized ergonomic gains could be offset by incompatibility with clinical workflow and institutional constraints.

In social care, reports of physical relief were tentative but present. Users noted postural improvements and reduced knee or shoulder discomfort, particularly among older staff [42, 43]. However, much of the relief was framed in conditional or relational terms, like useful “for some tasks” or “for some users.” Unlike in healthcare and industry, relief in social care was not framed in terms of productivity but rather as a support for continued caregiving.

Across industry, benefits were linked to productivity and task efficiency. In automotive, cleaning, agriculture, and retail subsectors, exoskeletons were frequently linked to physical relief, especially in overhead or static tasks. Quantitative data confirmed reductions in upper limb muscle activity: up to 34% in automotive assembly [26], and around 31% in cleaning [17]. Workers echoed these benefits like, shoulder fatigue reduced [12, 31], arm support felt beneficial [34], and “it could help with back strains from lifting, twisting, and turning” [13]. Yet, relief was often conditional. In retail and multiple-sector trials, some users described discomfort overriding benefit: “I did not feel pain in my back, but the exoskeleton was hindering me‥” [47]; relief came after removing the exoskeleton [49]. Discomfort from straps, shifting fit, and heat build-up was most common in rigid-frame designs. Back-support exoskeletons showed modest benefit in agriculture [45] and logistics [44], and improved posture in retail [23]. But again, these gains were often not sustained over time during tasks involving dynamic movements [28]. Several workers noted that relief alone wasn’t enough: devices needed to fit the task and tools [8, 11], otherwise acceptance waned.

#### 5.2.2 Comfort and usability: shaped by task, fit, and context

Comfort and usability emerged as critical influences on user experience across all three sectors, but the meaning and impact of discomfort somewhat varied by context. While brief relief was often reported early, discomfort commonly increased with time and interfered with routine use. Across all sectors, poor fit, weight, pressure points, and thermal discomfort were recurring barriers, yet how these issues shaped experience depended heavily on the nature of the work and setting.

In healthcare, comfort was strongly tied to professional performance, movement fluidity, and clinical hygiene. Surgeons and ICU staff noted early physical relief, but longer wear triggered discomfort at contact points, especially for smaller or female users [2, 3]. Rapid donning, sterile compatibility, and ease of movement were essential in fast-paced environments [3, 5, 40]. Nursing staff raised concerns about hygiene and appearing robotic, particularly in geriatric settings [6, 37]. However, with the exception of one long term study [37], most findings were based on short trials, making it difficult to assess long-term comfort in real workflows.

In social care, comfort was interpreted through the lens of hands-on caregiving in nonclinical, home-like environments, where ease of movement, unobtrusiveness, and hygiene in shared-use settings are more critical than clinical precision. Discomfort during squatting or showering, especially in thighs and shoulders, was noted, along with strap entanglement and hygiene concerns in shared-use settings [42, 43].

In industry, comfort was closely tied to task type, duration of use, heat exposure, and design specifics. While some workers reported comfort and support during static or repetitive tasks [12, 19, 14], discomfort often escalated over time due to heat, pressure, and rigid frames [14, 24, 30, 34]. In construction and manufacturing, strap pressure and mobility constraints were especially problematic for dynamic tasks [15, 20, 39]. Unlike healthcare, thermal discomfort, equipment interference, and bulk, not sterility or patient perception, were deterrents for sustained use [28, 34, 38]. A longer trial, unlike in healthcare, showed that discomfort could decrease with regular use [30], while others emphasized the importance of personal adjustability and morphological adaptability for comfort and acceptance [12, 31].

#### 5.2.3 Fit and inclusivity: persistent challenges with sectoral implications

Across all three sectors, fit and inclusivity emerged as baseline expectations, not as bonuses. Users expected exoskeletons to adapt to their bodies, roles, and environments. Where this alignment was missing, discomfort, exclusion, or withdrawal followed. Yet the implications of poor fit diverged by context.

Design inclusivity, particularly around gender and body size, emerged as a recurring barrier, especially in healthcare and industry. In the hospital setting, female participants were excluded due to fit issues [1], and one noted the need to upsize surgical gowns. Study 5 echoed this, with users reporting chest pressure and discomfort during dynamic care tasks.

Social care participants also noted fit-related discomfort [43], but it was described less as exclusion and more as a manageable irritation: “The device is not able to be used by diverse body types.” These findings suggest that design equity remains unresolved – with practical and symbolic implications. In settings like healthcare, a poor fit undermined both usability and perceived legitimacy. In the industry, it interrupted the workflow. In social care, it limited universality but did not appear to threaten user identity.

In industry, fit was the most consistently cited factor, shaping adoption and implementation. Users across manufacturing, agriculture, and retail noted issues with straps, pressure points, and inadequate adjustability, particularly for petite or smaller-bodied users [24, 30, 34, 45]. Industrial users in studies 22 and 36 described similar problems: “straps didn’t adjust right,” “chafing on hips,” and “not usable across all sizes.” Study 38 reported the withdrawal of smaller-bodied participants after a two-week trial due to poor fit. One participant withdrew from a long-term trial due to persistent discomfort and poor fit [30]. Rigid designs often fail to accommodate body variation, leading to chafing, restricted movement, and heat accumulation [34, 38, 44]. Where fit was achieved and support was felt, especially in repetitive or overhead tasks, adoption was higher [19, 30].

#### 5.2.4 Social and organizational climate shape acceptance

The uptake and perception of exoskeletons were shaped by organizational endorsement, peer norms, and local culture.

In healthcare, organizational support emerged as a strong determinant of uptake. In surgical settings, participants recommended using attending surgeons as implementation champions to promote team buy-in and ergonomic awareness [3]. Nurses associated positive experiences not just with comfort and movement, but with feeling socially at ease in front of others [37]. Organizational identity also played a role: in geriatric care, staff who saw deep meaning in relational aspects of their work reported a lower willingness to adopt exoskeletons [6]. Importantly, teams with visible leadership engagement were more likely to sustain interest and trust.

In social care, the study highlighted that managers may end up having new tasks related to guidance and monitoring. Here, gentle onboarding and nonintrusive trialing seemed to foster quiet acceptance with devices described as “unobtrusive” and “neutral” [43].

In the industry, organizational conditions vary. A shared experience within one company saw 61.5% of participants wishing to continue use [41], while cross-company comparison showed that culture shaped acceptance: Company A reported stronger positive effects than Company B [27]. Poor training emerged as a barrier, where one participant broke a device due to incorrect donning [47], while others reported discomfort linked to unfamiliarity and rushed onboarding [12, 39]. Structured, time-efficient training was seen as critical for early usability and confidence [41]. Voluntary use remained key, with experts warning that pressure to adopt could undermine trust [44].

#### 5.2.5 Meaning and identity: from ethical tension to symbolic neutrality

Perhaps one of the most context-dependent themes was the meaning users attributed to exoskeletons.

In healthcare, users expressed emotional ambivalence. In Study 6, some geriatric nurses feared that exoskeletons would appear robotic or depersonalizing, clashing with the relational nature of care. Perceived usefulness improved willingness to adopt, but concerns about role disruption and ethical acceptability still surfaced.

In social care, by contrast, exoskeletons were seen as socially neutral. Studies 42 and 43 noted that residents showed mild curiosity but little concern, and users reported no stigma or symbolic resistance. This neutrality may reflect the early stages of the trial.

In the industry, symbolic meaning was shaped more by peer norms than ethical concerns. Some workers in manufacturing and construction feared exoskeleton use might signal weakness [11, 13, 41]. However, across most industrial contexts, identity played a lesser role than practicality: rejection typically stemmed from discomfort or poor task fit rather than stigma or ethical conflict.

Rather than being universally helpful or harmful, exoskeletons are experienced through the lens of bodily relief, procedural fit, team norms, and personal meaning.

### 5.3 Objective 3: To identify factors influencing the adoption and implementation of exoskeletons in healthcare, social care, and industry

In line with the implementation science literature (Nilsen, 2015), we define barriers as factors that impede, hinder, or negatively influence the implementation and sustained use of an innovation. While facilitators are factors that enable, support, or positively influence implementation outcomes. They may operate at multiple levels, including the individual, organizational, and policy or system levels. A basic content analysis of the included studies identified key barriers and facilitators influencing exoskeleton adoption and implementation.

#### 5.3.1 Barriers to adoption and implementation

Barriers to exoskeleton adoption and implementation were most commonly identified through user and stakeholder feedback across all sectors. Physical discomfort, including pain, pressure, heat, and localized discomfort at contact points, was the most frequently reported challenge, often linked to prolonged use, design limitations, or specific tasks. Poor ergonomic fit and sizing, particularly for women or those with atypical body shapes, were persistent barrier, with many users describing difficulty achieving comfort or stable support. Movement restriction and device weight or bulk were also widely cited, especially when they interfered with the ability to perform regular duties, work in confined spaces, or adapt to dynamic tasks. Users frequently described task incompatibility and workflow disruption as major impediments, noting that exoskeletons often did not fit seamlessly into existing routines or required time-consuming adjustments that were impractical in fast-paced environments. Hygiene and infection control concerns, especially regarding shared devices or challenges with cleaning, were particularly salient in healthcare and social care but were also noted in industry. Usability and operational complexity, including difficulties with donning, doffing, or adjusting devices, were recurrent barriers that reduced willingness to persist with use. Finally, concerns around cost, maintenance, and uncertain cost/benefit were noted, especially in settings where the perceived value was unclear, or resources for training and upkeep were limited.

Overall, these barriers frequently co-occurred and were often interconnected: discomfort and poor fit undermined usability, while workflow and organizational constraints compounded practical challenges. Even when some benefits were recognized, persistent barriers limited widespread adoption and sustained use. A detailed breakdown of the most frequently reported barriers by sector is provided in Table 5.

**Table 5.** Main barriers to exoskeleton adoption and implementation by sector

Barrier theme	Definition	Healthcare studies	Industry studies	Social care studies
Physical discomfort, pain, or thermal burden	Discomfort, pain, pressure, heat/thermal issues, fit-related discomfort	1, 4–7, 37, 40	14–16, 18, 22, 24, 26, 28, 29, 30, 32, 34, 38, 39, 44, 45, 47, 49	42, 43
Poor ergonomic fit/lack of personalization	Device does not fit a range of body types or sizes, including gender/anthropometric mismatches	1, 4, 6, 7, 40	10–15, 18, 22, 25, 26, 29, 30, 32, 34, 44, 45, 47	43
Movement restriction/limited mobility	Device restricts range of motion, interferes with task execution or agility	2, 3, 6, 40	13–16, 18, 19, 22, 24, 26, 28, 29, 30, 39, 44, 45, 47, 49	42, 43
Device weight and bulk	Perceived as heavy or bulky, cumbersome for long use or specific tasks	2–4, 40	10, 11, 13–16, 18, 22, 24, 26, 29, 30, 32, 34, 39, 44, 45, 47, 49	Not reported
Task incompatibility/workflow disruption	Device does not suit job demands, slows workflow, or is impractical in real work	1, 3–6, 40	10, 12–16, 19, 20, 22, 24, 25, 26, 28–30, 32, 38, 39, 41, 44, 45, 47	42, 43
Hygiene/infection control	Concerns about cleanliness, sweat, shared use, or infection risk	37, 40	10, 11, 15, 32, 44, 45, 47	43
Usability/operational complexity	Difficult to use, don/doff, adjust, or learn (cognitive load); operationally complex	3, 5, 6, 37, 40	10, 11, 13, 14–16, 18, 22, 24, 25, 26–30, 32, 33, 34, 39, 41, 44, 45, 47, 49	43
Cost, maintenance, and uncertain cost–benefit	High initial cost, ongoing maintenance, doubts about value for money	3, 5, 40	13, 18, 38, 47	Not reported

#### 5.3.2 Facilitators of adoption and implementation

Facilitators of exoskeleton adoption and implementation were most often identified through direct user feedback, post-trial interviews, and stakeholder recommendations. The most widely cited facilitator was physical relief. Users frequently reported reductions in pain, fatigue, or musculoskeletal strain, as well as improved posture during specific tasks or after prolonged use. Task compatibility and usability also played a crucial role. Devices that are easy to don, doff, adjust, or seamlessly fit into existing workflows were consistently rated more favorably. Perceived usefulness and intention to use emerged as another key facilitator, with users expressing greater acceptance when they believed in the value or relevance of the device for their role or colleagues. Recommendations for future use, willingness to trial again, and advocacy for others were common among those who experienced clear benefits. Organizational and social support, including visible management backing, peer encouragement, effective training, and a positive workplace culture, can boost uptake and sustained use. Where trialing was voluntary and leadership was engaged, users described feeling more confident and supported in trying new technology.

Finally, design adaptability and adjustability, such as customizable fit, comfort features, or the ability to tailor the device to different tasks, were frequently mentioned as a facilitator. Opportunities for participation, codesign, and feedback further enhanced engagement, with many users emphasizing the value of being involved in the implementation process.

Taken together, these facilitators show that adoption is most likely when exoskeletons deliver tangible physical benefits, fit smoothly into established work patterns, and are supported by an inclusive, responsive implementation environment. A detailed breakdown of the most frequently reported barriers by sector is provided in Table 6.

**Table 6.** Main facilitator of exoskeleton adoption and implementation by sector

Facilitator theme	Definition	Healthcare studies	Industry studies	Social care studies
Physical relief (pain/fatigue reduction, improved posture)	Users experienced reduced pain, fatigue, or improved posture	1, 2, 4, 5–7, 37	8–11, 14, 18, 19, 21–26, 29, 30, 32, 34, 35, 38, 41, 44, 48, 49	42, 43
Task compatibility/usability	Device is easy to don/doff, adjust, use; suits specific tasks or environments	2–7, 37	8–10, 12–14, 18, 19, 21–27, 29, 30, 32, 34, 35, 38, 41, 44, 47, 48, 49	42, 43
Perceived usefulness/intention to use	Users believe in the benefit, would use device again, recommend for others	1–7, 40	8–14, 18, 19, 21–27, 29, 30, 32, 34, 35, 38, 41, 44, 47, 48, 49	Not reported
Organizational and social support	Support from management, peers, training, positive team culture, leadership	3, 6, 7, 37, 40	11, 21, 27, 30, 38, 41,44, 45, 47	43
Design adaptability/adjustability	Device can be personalized/adjusted for comfort, fit, and task	1, 2, 4–7, 37	8–14, 18, 19, 21, 22–27, 29, 30, 32, 34, 35, 38, 41, 44, 47, 48, 49	43
Participation, codesign, and positive implementation process	User involvement in selection, trialing, codesign, and ongoing feedback	3, 5, 6, 40	13, 18, 19, 21, 24, 25, 27, 30, 33, 34, 35, 41, 44, 45, 47	43
Wider value and social acceptance	Benefits for diverse users, perceived status, and social or professional value	6, 37, 40	10–13, 24, 27, 30, 41, 44	42, 43

#### 5.3.3 Conceptual synthesis of findings

**Figure 2.**
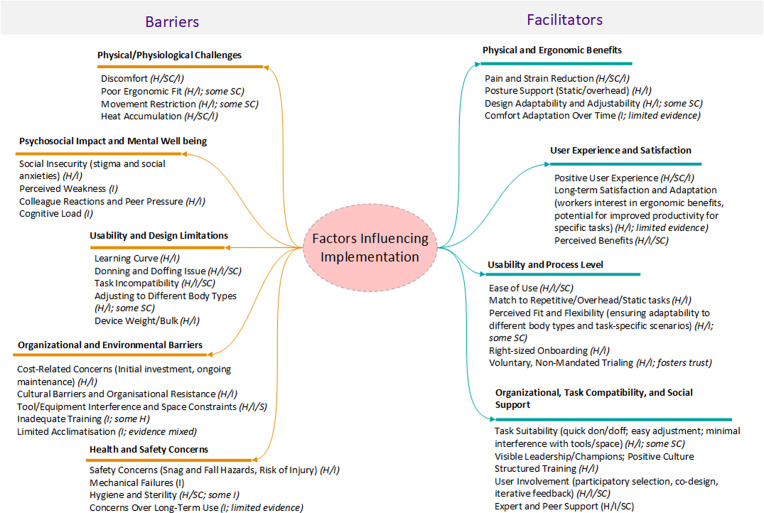
Conceptual map of factors influencing exoskeleton adoption and implementation in occupational settings. H = Healthcare; SC = Social care; I = Industry.

To support interpretation of the barriers and facilitators identified in this review, we developed a conceptual map (Figure 2) that synthesizes findings from both the thematic analysis of user experiences (Objective 2) and the content analysis of adoption and implementation factors (Objective 3). This map organizes barriers and facilitators into broader domains operating at the individual, task, and organizational levels, offering a cross-sectoral lens to understand exoskeleton implementation challenges and enablers.

## 6. Discussion

This novel scoping review synthesized evidence from forty-nine studies on exoskeleton use across healthcare, social care, and industrial sectors, using three distinct yet interlinked objectives. By focusing on user experiences, adoption barriers, and facilitators, this review provides a cross-sectoral understanding of current practices and identifies key gaps for future research. Studies reported ergonomic benefits; however, the effectiveness of different exoskeleton models and levels of user acceptance varied across sectors. These variations were influenced by factors such as comfort, adaptability, task compatibility, and the specific demands of different occupational environments.

### 6.1 Device design: passive dominance, active potential

Passive exoskeletons dominate field-based studies, aligning with existing literature that highlights their advantages, such as being lighter, cheaper, and mechanically simpler than active devices. However, as highlighted by Sawicki et al. (2020), passive devices provide fixed mechanical properties, making them more suited for repetitive, habitual tasks like walking or lifting at a consistent pace. This may limit their adaptability in dynamic environments, such as health and social care, where tasks frequently change, and require greater responsiveness. For example, Farah et al. (2023) pointed out that while passive exoskeletons are beneficial in reducing musculoskeletal strain for industrial tasks, their effectiveness diminishes in settings like nursing, where frequent lifting, patient mobility, and different physical tasks demand a more adaptive approach. In contrast, active exoskeletons offer the flexibility of customized torque profiles that can adjust in real time to support dynamic tasks. However, their adoption is hindered by bulky motors and high energy requirements, which may reduce usability in real-world settings. Therefore, future research should focus on testing and refining these devices for real-world implementation, particularly in sectors with high physical demands. Adult social care workers, for example, represent an under-researched group facing physical challenges. Studies from Finland (Saurio et al., 2023) have explored exoskeleton applications in adult social care, but further investigation is needed to address the specific needs of care workers, a crucial workforce regularly performing demanding physical tasks. Although the included studies were conducted in workplace environments, a large number used simulated or controlled settings, particularly in healthcare. In healthcare, controlled environments, such as operating rooms, allow structured simulations or controlled testing with workers, which may explain why healthcare studies are conducive to workplace-focused exoskeleton trials, despite comparatively fewer studies overall. Also, the acute ergonomic needs in healthcare, such as reducing strain during patient handling or prolonged standing, make these settings conducive to exoskeleton trials. However, the limited ecological validity of findings from controlled settings highlights the need for more empirical research conducted in dynamic, real-world work environments.

### 6.2 Methodological gaps and research fragmentation

Our review found an upward trend toward field-based studies, reflecting researchers’ growing response to calls for improved ecological validity (Crea et al., 2021). Despite this progress, methodological inconsistencies persist, echoing the concerns raised by De Bock et al. (2022) about the fragmentation in exoskeleton research. Most studies still focus on short-term use, with limited evidence on the sustained impact of exoskeletons on user health and productivity. However, a few exceptions exist that together represent an 18-month longitudinal evaluation of exoskeleton use in automotive assembly (Kim et al., 2021, 2022). These studies, along with one from healthcare (Farah et al., 2023), provide rare insights into longer-term usability, adaptation, and implementation challenges in real-world conditions. While short-term data offers valuable insights into immediate user experiences, addressing longer durations of wear will be critical for ensuring the practical utility and adoption of exoskeletons in real-world environments. The majority of evaluations reported a trial duration of ≤2 hours, primarily for usability and acceptance testing. These findings align with Hensel and Keil (2019), who observed discomfort increasing after 2–4 hours of continuous wear. These issues reflect the complexity of the device design, which, while intended to alleviate strain, can introduce physical discomfort if not properly fitted or tailored to task-specific needs. This reflects a longstanding concern: discomfort was highlighted as a significant challenge in early evaluations, such as those by de Looze et al. (2016), who cautioned that even minimal discomfort at the user–device interface could hinder acceptance. This persistent concern, despite successive device iterations, raises important questions about whether discomfort remains an unresolved barrier across settings. The findings highlight the importance of evaluating exoskeletons across varying durations and settings to better understand their real-world usability and ergonomic implications. One potential approach is a more flexible usage model, in which exoskeletons are worn intermittently during high-load or repetitive tasks and removed thereafter. Lucia Botti and Melloni (2024) support this strategy, noting that such task-specific use can help reduce discomfort associated with prolonged wear and improve user acceptance. While this approach is gaining traction in industrial contexts, it remains underexplored in healthcare and social care environments, where tasks are more varied and relational. In this review, studies also highlight declining user acceptance over time due to fit issues, hygiene burdens, thermal discomfort, or restricted mobility (Riemer and Wischniewski, 2023; Wioland et al., 2024). This declining acceptance may also contribute to the scarcity of longitudinal studies, as maintaining participant engagement becomes increasingly difficult. While Kim et al. (2021) observed variability in user experiences over extended periods, their findings showcase the importance of sustained ergonomic benefits and usability in promoting long-term adoption.

Measurement tools were also highly varied. While EMG, the Borg CR10, and the SUS were common, few studies justified their selection or triangulated results with longer-term outcome data. Broader psychosocial and organizational variables, including user satisfaction over time, behavioral adaptation, or social identity impacts, were rarely assessed. Furthermore, small sample sizes and nonrepresentative participant demographics (e.g., gender imbalances and industry-specific biases) limit the generalizability of findings.

### 6.3 Sector-specific barriers

Factors like social acceptance, perceived aesthetics, and emotional comfort, such as how users feel about wearing the exoskeletons in front of colleagues or patients, are often overlooked in the focus on ergonomic benefits. These factors are particularly critical in social care settings, where caregiving involves not just physical tasks but also emotional connection and trust. The visibility or bulkiness of exoskeletons can disrupt these interactions, potentially leading to perceptions of depersonalization or distancing between care workers and recipients. Similarly, in healthcare, while task efficiency and ergonomic relief are prioritized, patient-facing roles may still encounter challenges if the devices are perceived as intrusive or incompatible with the caregiving environment. Saurio et al. (2023) highlighted that the emotional and social factors, particularly in caregiving roles, can outweigh the perceived ergonomic benefits, leading to resistance despite initial positive feedback. Hence, unique to this review, emotional barriers in patient-facing environments emerged as a challenge. The implications of these findings mean that exoskeletons ought to be designed and developed in a manner that goes beyond physical support to integrate seamlessly into the social and emotional dynamics of the workplace. Additionally, in industry, consistent with the findings from Elprama et al. (2023), symbolic meanings were more often shaped by peer norms and productivity concerns. Diverging expectations between workers (who value relief) and managers (who prioritize performance) may result in misaligned implementation goals. This contrast reinforces the need for inclusive, collaborative implementation processes and training.

Our review advances the field by offering cross-sectoral insights into exoskeleton adoption, highlighting shared and sector-specific barriers and facilitators across healthcare, social care, and industry. While prior research has explored acceptance in industrial contexts, such as Elprama et al.’s (2022), our findings underscore additional considerations in caregiving environments, where emotional, relational, and contextual factors play a critical role. Related reviews ( e.g., Ali et al., 2021), have mapped biomechanical and technical barriers, such as load reduction, fit, and comfort, primarily in industrial settings. Building on this, our review expands the lens to include less-explored emotional and symbolic barriers that shape uptake in patient and client facing roles. Such differences reinforce the need for context-sensitive implementation strategies. Although exoskeletons are perceived as effective in alleviating physical strain, their performance remains highly context-dependent, influenced by factors such as workplace practices, organizational cultures, and task-specific demands. These contextual elements highlight the need for further research that prioritizes real-world applications to optimize exoskeleton use. Addressing these adoption and implementation barriers might require multi-level strategies that combine clear communication, collaborative decision-making, and well-structured training programs. While immediate ergonomic relief drives adoption, particularly in high-injury sectors such as healthcare and construction, the extent to which these benefits translate into long-term outcomes remains underexplored. These findings indicate that immediate ergonomic benefit alone does not ensure sustained adoption. For exoskeletons to be successfully implemented, they must align with users daily routines, perceived needs, and social environments. This highlights the importance of inclusive design, meaningful user engagement, and organizational readiness as critical enablers of long-term adoption and integration.

### 6.4 Strengths and limitations

A key strength of this review is that it followed the latest JBI Scoping Review methodology, which offers a structured and rigorous framework for systematically mapping existing evidence (Peters et al., 2021; Pollock et al., 2023). This review incorporated content analysis and a systematic classification of factors influencing the adoption and implementation of exoskeletons. This led to the unique contribution of this review by the development of a conceptual map that synthesizes findings, offering a tool for researchers and practitioners alike. However, there are limitations to scoping review methodologies. Following JBI guidelines, no critical appraisal or risk of bias assessments were conducted, which limits the ability to assess the methodological quality of the included literature (Munn et al., 2018). This approach is appropriate for a scoping review, where the primary aim is to capture a broad overview of the existing evidence rather than exclude papers based on methodological rigor. By including studies based on their relevance to the review objectives, our scoping review ensures a complete mapping of evidence, though it may affect the reliability of identified themes. Future systematic reviews could build on these findings by incorporating critical appraisals to assess study quality.

Moreover, excluding gray literature and reviews narrowed the scope of the review, potentially missing practice-based evidence or insights from non-published sources. The decision ensured manageability, prevented duplication, and maintained methodological consistency. Besides, although the search strategy was developed with guidance from a research librarian, it relied on predetermined keywords and subject headings, meaning some relevant studies may not have been captured. Nevertheless, the broad database coverage helped mitigate this limitation. Finally, while the structured coding and reporting framework ensured consistency in categorizing themes within the conceptual map, reliance on findings, discussions, and conclusions across the literature with varying methodological rigor, often based on short-duration, subjective evaluations, may still introduce interpretive bias.

## 7. Conclusion

This novel scoping review maps the evolving evidence base on exoskeleton use in healthcare, social care, and industry, indicating a growing interest in their application for manual handling tasks, particularly in roles involving repetitive lifting and sustained postures. However, their effectiveness and acceptance remain highly context-dependent, with sector-specific challenges influencing adoption, such as sterility in clinical settings, emotional labor in social care, and task variability in industry, which shape uptake. Exoskeletons reported ergonomic benefits in reducing physical strain, particularly in static and repetitive tasks, their usability in dynamic environments, such as patient handling and social care, remains underexplored. Future research should prioritize sector-specific, real-world implementation studies that examine usability and comfort across diverse work routines, not just isolated task trials. Comparative, longitudinal studies across sectors could also shed light on how acceptance evolves over time across different organizational and cultural conditions. Participatory and codesign approaches, involving end users and managers will be essential to ensure contextual fit and sustained adoption. To advance implementation science in this field, the exoskeleton research community should adopt theoretically informed, multilevel frameworks such as Normalization Process Theory (NPT) (May and Finch, 2009) or the Consolidated Framework for Implementation Research (CFIR) (Damschroder et al., 2009), to explore how individual experience, organizational readiness, and wider system factors interact. Addressing these challenges will be essential to ensure that exoskeletons are not only ergonomically beneficial but also meaningfully align with real-world occupational needs.

### 7.1 Implications for future research and practice

While specific practice recommendations for exoskeleton use in healthcare, social care, and industry require further empirical validation, this review offers preliminary insights that may inform future adoption and implementation strategies. The findings of this review highlight the need for longitudinal, field-based studies to assess the sustained impact of exoskeletons on worker health, productivity, and long-term usability across healthcare, social care, and industry sectors. Most existing research remains short-term, limiting understanding of real-world implementation and effectiveness. Upcoming research should also explore the interplay between individual, organizational, and contextual factors in greater depth to understand practicalities for implementation. Future implementation research would benefit from the adoption of sector-relevant, standardized assessment frameworks such as the Exoworkathlon (Kopp et al., 2022), to enable more robust comparisons and accelerate knowledge translation across sectors. For practitioners, this review presents the importance of organizational readiness and supportive environments for fostering exoskeleton adoption. Cross-sectoral collaboration may further drive innovation, ensuring that exoskeletons evolve to meet the diverse demands of healthcare, social care, and industrial applications.

## Supporting information

Bhat et al. supplementary material 1 Bhat et al. supplementary material

Bhat et al. supplementary material 2 Bhat et al. supplementary material

## Data Availability

The authors confirm that the data supporting the findings of this study are available within the article and its supplementary materials.

## Supplementary material

The supplementary material for this article can be found at http://doi.org/10.1017/wtc.2025.10033.
