# Supplementary material for: Mapping the evidence on occupational exoskeleton use for the workforce in healthcare, social care, and industry: A systematic scoping review
Source: Wearable Technol. 2025 Oct 28;6:e53. doi: 10.1017/wtc.2025.10033 (PMC12569392; doi:10.1017/wtc.2025.10033)
Supplement: Bhat et al. supplementary material 1 — Bhat et al. supplementary material [file S2631717625100339sup001.pdf]

Appendix1 – Search Strategy

Data base	WoS	
#	Search terms	Hits
1	TS=(workforc* OR "work* force*" OR workplac* OR "work* place*" OR staff* OR employe* OR worker* OR occupation* OR profession* OR Manpower* OR "Work* group*" OR "Workgroup*" OR "work* team*" OR "Work* pool*" OR "Work* unit*" OR "workunit*" OR "Occupation* activit*" OR lift* OR carry* OR push* OR pull* OR task* OR "occupational activit*" or TS=(Manual* near/2 (labo$r* OR handl* OR move* OR moving OR transport* OR carry* OR work* OR operation*)) or TS=(Heavy near/2 (labo$r* OR handl* OR move* OR moving OR transport* OR carry* OR work* OR operation*)) or TS=(Physical* near/2 (labo$r* OR handl* OR move* OR moving or transport* OR carry* OR work* OR operation*)) or TS=(Material* near/2 (labo$r* OR handl* OR move* OR moving OR transport* OR carry* OR work*)) or TS=(Object* near/2 (handl* OR move* OR moving OR transport* OR carry* OR work* OR operation*)) or TS=(Load* near/2 (handl* OR move* OR moving OR transport* OR carry* OR work* OR operation*)) or TS=(Heath* near/2 (home* OR work* OR care* OR labo$r* OR handl* OR move* OR moving OR transport* OR carry* OR work* OR operation*)) or TS=(Occupation near/2 (handl* OR move* OR moving OR transport* OR carry*))	4,817,557
2	TI= (exoskeleton* OR exosuit* OR "hybrid assistive limb*" OR "wearable robot*")	6,169
3	TS= (Health* NEAR/4 care* OR *care* OR "care support" OR Occupation* OR industr* or "social near/4 care")	4,722,340
4	1 and 2 and 3	401
Data base	SCOPUS	
#	Search terms	hits
1	(TITLE-ABS-KEY (workforc* OR "work* force*" OR workplac* OR "work* place*" OR staff* OR employe* OR worker* OR occupation* OR profession* OR manpower* OR "Work* group*" OR "Workgroup*" OR "work* team*" OR "Work* pool*" OR "Work* unit*" OR "workunit*" OR "Occupation* activit*" OR lift* OR carry* OR push* OR pull* OR task* OR "occupational activit*") OR ("Manual*" W/2 (labour* OR handl*	8,069,772

	OR move* OR moving OR transport* OR carry* OR work* OR operation*)) OR ("Heavy" W/2 (labour* OR handl* OR move* OR moving OR transport* OR carry* OR work* OR operation*)) OR ("Physical*" W/2 (labour* OR handl* OR move* OR moving OR transport* OR carry* OR work* OR operation*)) OR ("Material*" W/2 (labour* OR handl* OR move* OR moving OR transport* OR carry* OR work* OR operation*)) OR ("Object*" W/2 (handl* OR move* OR moving OR transport* OR carry* OR work* OR operation*)) OR ("Load*" W/2 (handl* OR move* OR moving OR transport* OR carry* OR work* OR operation*)) OR ("Heath*" W/2 (home* OR work* OR care* OR labour* OR handl* OR move* OR moving OR transport* OR carry* OR work* OR operation*)) OR ("Occupation" W/2 (handl* OR move* OR moving OR transport* OR carry* OR work*)))	
2	TITLE (exoskeleton* OR exosuit* OR "hybrid assistive limb*" OR "wearable robot*")	**7,789**
3	TITLE-ABS-KEY (health* W/4 care* OR *care* OR "care support" OR occupation* OR industr* OR "social W/4 care")	**2,476,953**
4	1 and 2 and 3	**95**
Data base	**Medline**	
#	**Search terms**	**hits**
1	TX workforc* OR "work* force*" OR workplac* OR "work* place*" OR staff* OR employe* OR worker* OR occupation* OR profession* OR manpower* OR "Work* group*" OR "Workgroup*" OR "work* team*" OR "Work* pool*" OR "Work* unit*" OR "workunit*" OR "Occupation* activit*" OR lift* OR carry* OR push* OR pull* OR task* OR "occupational activit*") OR ("Manual*" N2 (labour* OR handl* OR move* OR moving OR transport* OR carry* OR work* OR operation*)) OR ("Heavy" N2 (labour* OR handl* OR move* OR moving OR transport* OR carry* OR work* OR operation*)) OR ("Physical*" N2 (labour* OR handl* OR move* OR moving OR transport* OR carry* OR work* OR operation*)) OR ("Material*" N2 (labour* OR handl* OR move* OR moving OR transport* OR carry* OR work* OR operation*)) OR ("Object*" N2 (handl* OR move* OR moving OR transport* OR carry* OR work* OR operation*)) OR ("Load*" N2 (handl* OR move* OR moving OR transport* OR carry* OR work* OR operation*)) OR ("Heath*" N2 (home* OR work* OR care* OR labour* OR handl* OR move* OR moving OR transport* OR carry* OR work* OR operation*)) OR ("Occupation" N2 (handl* OR move* OR moving OR transport* OR carry* OR work*	**3,029,947**
2	TI exoskeleton* OR exosuit* OR "hybrid assistive limb*" OR "wearable robot*"	**1,963**

3	TX Health* N4 care* OR *care* OR "care support" OR Occupation* OR industr* or "social N4 care"	**5,300,196**
4		**238**
Data base	**PyschInfo**	
#	**Search terms**	**hits**
1	TX workforc* OR "work* force*" OR workplac* OR "work* place*" OR staff* OR employe* OR worker* OR occupation* OR profession* OR manpower* OR "Work* group*" OR "Workgroup*" OR "work* team*" OR "Work* pool*" OR "Work* unit*" OR "workunit*" OR "Occupation* activit*" OR lift* OR carry* OR push* OR pull* OR task* OR "occupational activit*") OR ("Manual*" N2 (labour* OR handl* OR move* OR moving OR transport* OR carry* OR work* OR operation*)) OR ("Heavy" N2 (labour* OR handl* OR move* OR moving OR transport* OR carry* OR work* OR operation*)) OR ("Physical*" N2 (labour* OR handl* OR move* OR moving OR transport* OR carry* OR work* OR operation*)) OR ("Material*" N2 (labour* OR handl* OR move* OR moving OR transport* OR carry* OR work* OR operation*)) OR ("Object*" N2 (handl* OR move* OR moving OR transport* OR carry* OR work* OR operation*)) OR ("Load*" N2 (handl* OR move* OR moving OR transport* OR carry* OR work* OR operation*)) OR ("Heath*" N2 (home* OR work* OR care* OR labour* OR handl* OR move* OR moving OR transport* OR carry* OR work* OR operation*)) OR ("Occupation" N2 (handl* OR move* OR moving OR transport* OR carry* OR work*	**1,745,547**
2	TI exoskeleton* OR exosuit* OR "hybrid assistive limb*" OR "wearable robot*"	**325**
3	TX Health* N4 care* OR *care* OR "care support" OR Occupation* OR industr* or "social N4 care"	**1,013,408**
4	1 and 2 and 3	**45**
Data base	**CINAHL**	
#	**Search terms**	**hits**

**1**	TX workforc* OR "work* force*" OR workplac* OR "work* place*" OR staff* OR employe* OR worker* OR occupation* OR profession* OR manpower* OR "Work* group*" OR "Workgroup*" OR "work* team*" OR "Work* pool*" OR "Work* unit*" OR "workunit*" OR "Occupation* activit*" OR lift* OR carry* OR push* OR pull* OR task* OR "occupational activit*") OR ("Manual*" N2 (labour* OR handl* OR move* OR moving OR transport* OR carry* OR work* OR operation*)) OR ("Heavy" N2 (labour* OR handl* OR move* OR moving OR transport* OR carry* OR work* OR operation*)) OR ("Physical*" N2 (labour* OR handl* OR move* OR moving OR transport* OR carry* OR work* OR operation*)) OR ("Material*" N2 (labour* OR handl* OR move* OR moving OR transport* OR carry* OR work* OR operation*)) OR ("Object*" N2 (handl* OR move* OR moving OR transport* OR carry* OR work* OR operation*)) OR ("Load*" N2 (handl* OR move* OR moving OR transport* OR carry* OR work* OR operation*)) OR ("Heath*" N2 (home* OR work* OR care* OR labour* OR handl* OR move* OR moving OR transport* OR carry* OR work* OR operation*)) OR ("Occupation" N2 (handl* OR move* OR moving OR transport* OR carry* OR work*	**1,844,013**
**2**	TI exoskeleton* OR exosuit* OR "hybrid assistive limb*" OR "wearable robot*"	**573**
**3**	TX Health* OR care* OR Occupation* OR industr* OR "social care"	**5,490,463**
**4**	1 and 2 and 3	**215**
	All Databases	**994**
